# Review Department

**Published:** 1890-11

**Authors:** 


### REVIEW DEPARTMENT.

THE PHILOSOPHY OF TUMOR DISEASE; A RESEARCH FOR PRINCIPLES OF ITS TREATMENT. By C. PITFIELD, Member of the Royal College of Surgeons, England; Author of "Dissolution and Evolution," and "The Science of Medicine," etc. London: Williams & Morgate. New York: G. P. Putnam's Sons. 1890.

The author of this interesting volume has already made a very valuable contribution to current medical literature, which was reviewed at the time in the pages of the JOURNAL, and which was regarded both in England and America as a distinct confirmation of the essential features of the doctrine of evolution. In the present work the author pushes his inquiries along the same lines of investigation, and has made an ingenious application of the principle laid down by the evolutionists, that the primordial form of organic increase is the generative process shown in the lowest living creatures. Rejecting the doctrine that tumors are the result of excessive cell development left behind in the shape of embryonic remains, he proceeds to point out their obvious and fundamental character of self-dependence. They do not obey the same laws of growth and decay as the normal tissues of the body. They have no functional value in the system, and while deriving their nutrition from the same source, they lead independent lives. The explanation of this self-dependence is the keynote to the hypothesis propounded by Mr. Mitchell. He recognizes two modes of increase in the organic world, continuous and discontinuous. Continuous increase is that by which organized beings grow to maturity; discontinuous is the reproduction by generation of new individuals by the same species. Looking backward in the light which evolution furnishes, we know that discontinuous increase marks the origin of organic life, for protozoa and prophyta are unicellular bodies, and multiply by division into similar cells having the same independent existence, just as multicellular organisms divide, with this difference, however, that the latter instead of dispersing, become integral portions of more highly organized beings. An interesting question here presents itself for solution, viz.: How the transition is effected from these unicellular protozoa to the multicellular metazoa? At this point we are left to conjecture, for however ingeniously the philosophy of evolution may answer the question, it can do so only by going back of facts. It supposes that masses of these simple cells became entangled in what is called an "aggregation plasmodium," which marks the first step toward feeble integration and consequent differentiation. The manner, therefore, in which protozoa lend themselves to the production of metazoa may be regarded as the earliest instance of continuous growth. This much granted, it is easy to conceive how differentiation may progress till we reach the higher forms of life, where continuous growth is effected by cell multiplication, resulting in the production of the various tissues that enter into the constitution of complex organisms. Be it observed that cell production is accomplished in the same way in unicellular and multicellular beings, and that the difference in the result begins where the latter lend themselves to differential histogenesis. The cells from which tumors are formed, having no *raison d' etre* in the bodily organism in which they appear, begin to act in accordance with the laws that govern their prototypes in the protozoa, *i. e.*, they revert to an ancestral mode of reproduction and remain homogeneous in character. Such, in very few words, is the substance of Mr. Mitchell's hypothesis concerning the growth and origin of tumors, and it must be said that the author has exhibited a most praiseworthy ingenuity in the construction of his theory, inasmuch as he has made it entirely comformable to facts, instead, as most theorists do, of bending facts to the support of their hypothesis. But there are other facts in primordial histogenesis which Mr. Mitchell, with equal ingenuity, invokes in support of his opinion. The most interesting of these is *histogenic dissolution*. In the simplest forms of life propagation is accomplished by the birth of new cells at the expense of the parent ones, the latter surrendering their lives to their successors.

In all known forms of bacilli this is the case, and even traces of the phenomenon may be observed in higher forms of life. An interesting instance of it may be witnessed in trees that have been ringed or have been subjected to some growth-retarding influence. Instead of continuing to grow they begin to flower, evincing thereby a tendency to prepare for the perpetuation of its species as soon as the incipient signs of impending death became manifest. May we not behold a similar reversion in the case of executed criminals whose violent taking off is usually accompanied by seminal emissions? But it is only in the case of protozoa that this phenomenon is constant and invariable. In the case of cells that contribute to the building up of the normal tissues of the body, the parent cell continues to live and undergoes histogenic metamorphosis rather than dissolution. Tumor cells exhibit this archaic tendency to dissolve as they give birth to new ones, and again this interesting fact of histogenic dissolution gives a warrant to the conclusion that they are the result of discontinuous growth, and to enjoy the autonomy that is characteristic of them. The author here forestalls an objection that might be raised against his hypothesis and which proceeds from the fact that the normal tissues would resist the encroachment of cells whose aggregate development would militate against their own well-being, unless it were allowed that they were identical with themselves in all respects except that they multiplied to excess. He admits, indeed, that such resistance does take place, and successfully, too, on the part of cells possessing the vigor which they exhibit in healthy tissues, and that it is only when they have entered upon the path of regeneracy that they yield to the intruding parasite. He upholds this conclusion by many references throughout his book to the incipient breaking down of tissues contiguous to growing tumors. In the chapters on morphology the author adduces a large number of instances in proof of the statement that tumor cells make their appearance at the moment when contiguous tissues manifest signs of breaking down. The examples of this occurrence, gathered from the pages of Waldeyer, Birchfield and Cooke, are interesting and instructive. They show that the conversion of impaired normal tissue may readily lead to cancer in nearly all the organs of the body, notably in the skin, clitoris, penis, sub-maxillary glands and stomach. And it is for the same reason that cicatricial surfaces are more prone to degeneration of this sort, since it is evident that their vital powers have been impaired by injury, and so they are left in a much more favorable condition to undergo retrogressive metamorphosis.

Mr. Mitchell devotes many pages of his interesting work to a consideration of the morphological character of tumors with the view of lending additional confirmation to his hypothesis. In treating of the modes of growth of tumors and their relations to surrounding parts, he sees excellent reason for concluding that neoplasia is a reversion to the genesis of unicellular organisms.

It is not to be supposed that though the greater part of Mr. Mitchell's work is devoted to the establishment of a theory, that it possesses no practical value. On the contrary, he assures us that his ulterior aim in maintaining his views is to deduce practical conclusions from them, and to strengthen the hands of the general practitioner in dealing with this difficult class of ailments. Accordingly, he has devoted the concluding portion of his work to practical references from his hypothesis, and treats his readers to many interesting and instructive statements.

On the whole, it may be said that Mr. Mitchell has done good service to the cause of scientific medicine by giving to the world his treatise on the "philosophy of tumor formation."

C. M. O'L.

---

A TEXT-BOOK OF COMPARATIVE PHYSIOLOGY FOR STUDENTS AND PRACTITIONERS OF COMPARATIVE (VETERINARY) MEDICINE. By WESLEY MILLS, M.A., M.D., D.V.S., Professor of Physiology in the Faculty of Human Medicine and the Faculty of Comparative Medicine and Veterinary Science of McGill University, Montreal; Author of a Text-Book of Physiology, &c. New York: D. Appleton & Co. London: Caxton House, Pater Noster Square. 1890.

This volume represents in a condensed shape the contents of Dr. Mills' larger work on "Animal Physiology," published last year, and reviewed on these pages at the time. The intent of the work is to furnish veterinary students and practitioners with the physiological data indispensable to the intelligent prosecution of their professional work. So far it admirably serves its purpose, and would be a source of interest and instruction to the general reader were it not that the author frequently sacrifices clearness of statement to a too exacting sense of conciseness. This is particularly noticeable in the introductory chapter on biology and cell development. Yet the interest that attaches to the subject is such that the student who desires to aequire much valuable information should not be debarred by this consideration from a careful perusal of these chapters. Dr. Mills has said almost all that is worth saying on the doctrine of biology viewed from the standpoint of evolution, and it is worth while bearing in mind the fact that the Spencerian doctrine furnishes the keynote to modern physiology. Touching the methods which modern science employs to substantiate ils conclusions, Dr. Mills has many beautiful and interesting things to say, and his illustrations wonderfully aid the text. *En passant*, I would remark that the graphic method he so well describes is open to one abuse, viz. : that it inclines to give the student a merely mechanical view of vital processes. All mechanical appliances having for object the elucidation of physiological functions should be employed with the restriction that they are intended to partially illustrate processes that have their origin in life, and which thus outlie the possibility of complete explanation by inert matter. The circulation of the blood and respiration are evidences of this truth. If the heart were a mere pumping machine its capacity for work would be speedily exhausted, and its break down under valvular trouble would be much more speedier than we find it to be. It is in the study of muscle physiology, especially, that the graphic method is most applicable, and the student will find in this chapter a most valuable aid to the understanding of this important phenomenon. The chapter on digestion is clear and exhaustive, and supplies to the student such knowledge as is indispensable regarding this delicate process. It is to the student of comparative physiology, especially, that this chapter is fraught with interest. Indeed, the student of human physiology may here learn the most useful of lessons, for without an adequate knowledge of the digestive function as it takes place in the lower animals the more complex process of human digestion will only be imperfectly understood. In no organ is the gradual development from the simple sac of primitive forms of life to the complicated structure of the mammal stomach more apparent than in the results of digestion as described by Dr. Mills. This hand-book of comparative physiology is a welcome contribution to didactic literature, and deserves the warmest commendation from those who are engaged in teaching the science of physiology.

C. M. O'L.

